# Colorectal tumour BRAF V600E and MLH1 promoter methylation status in the assessment of mismatch repair gene sequence variants of unknown clinical significance

**DOI:** 10.1186/1897-4287-10-S2-A75

**Published:** 2012-04-12

**Authors:** M Parsons, B Thompson, D Goldgar, J Hopper, M Jenkins, D Buchanan, J Young, A Spurdle

**Affiliations:** 1 Queensland Institute of Medical Research, Brisbane, Australia; 2 Department of Dermatology, University of Utah, Salt Lake City, USA; 3 Centre for Genetic Epidemiology, University of Melbourne, Melbourne, Australia

## 

Lynch Syndrome is the most common hereditary cause of colorectal cancer, caused by pathogenic mutations in the mismatch repair (MMR) genes that result in functional defects in the DNA mismatch repair complex. Individuals identified with a pathogenic mutation are at high-risk of early onset colorectal and/or endometrial tumours. However, up to 50% of MMR sequence variants are reported to be of unknown clinical significance, creating a problem for clinicians and patients.

We are developing a multifactorial model to assess the pathogenicity of MMR gene sequence variants of unknown clinical significance. Currently this model utilizes data on tumour microsatellite instability (MSI) and family segregation analysis to assess the likelihood that a variant carrier demonstrates features expected for a carrier of a pathogenic mutation. Tumour *BRAF* V600E mutation and *MLH1* promoter methylation are reported to be associated with MSI-H, sporadic colorectal cancer and may thus provide a strong prediction that a tumour with MSI-H status is not from a MMR gene mutation carrier.

A literature review of *BRAF* V600E and *MLH1* promoter methylation in colorectal cancer patients was performed. Frequency of these tumour features in each study was assessed, stratified by MSI status and reported/likely MMR gene mutation status. Frequency of these characteristics was also analysed in the Colon CFR dataset, and used to estimate a likelihood ratio (LR) of pathogenicity for use in multifactorial modelling.

As expected, the literature review of 4655 individuals from 33 studies revealed a high frequency of *BRAF* mutations in MSI-H tumours without a MMR mutation, which increased for the subset of cases with lack of MLH1 tumour protein expression. However, *BRAF* V600E mutation was identified in one patient with a PMS2 mutation, challenging previous assumptions that *BRAF* mutation status excludes positive MMR gene mutation status. *MLH1* promoter methylation data generated using a variety of experimental designs were available for only 2984 individuals from 40 studies, with inconsistencies in frequency between studies. Information was reported on methylation status of 204 known *MLH1* gene mutation carriers.

Analysis of the Colon CFR dataset showed that *BRAF* tumour mutation status predicted MMR gene mutation status when combined with tumour MSI-H status. However, MSI-L and MSS with *BRAF* did not provide any additional predictive power over MSI-L or MSS status alone. The likelihood of a tumour with MSI-H and *BRAF* V600E positive status to be from a non-carrier was 11.55 and 9.85 for population-based and clinic-based patients, respectively. However, 4/98 (4.08%) of MSI-H *BRAF* positive tumours were from MMR mutation carriers.

In conclusion, positive *BRAF* tumour status is not a conclusive test to exclude MMR gene mutation status for patients, but *BRAF*-MSI tumour status is a powerful addition to multifactorial models evaluating clinical significance of MMR gene variants.

